# A Novel Poly(ε-Caprolactone)-Based Photo-Crosslinkable Liquid Copolymer as a Versatile Drug Delivery Platform

**DOI:** 10.3390/pharmaceutics16111380

**Published:** 2024-10-27

**Authors:** Marcus Flowers, Nicole Mertens, Amanda Billups, Brenda M. Ogle, Chun Wang

**Affiliations:** Department of Biomedical Engineering, University of Minnesota, 7-105 Hasselmo Hall, 312 Church Street S.E, Minneapolis, MN 55455, USA; flowe105@umn.edu (M.F.); merte223@umn.edu (N.M.); billu024@alumni.umn.edu (A.B.); ogle@umn.edu (B.M.O.)

**Keywords:** biodegradable polymer, poly(ε-caprolactone), photo-crosslinking, controlled release

## Abstract

**Background/Objectives**: Hydrophobic semi-solid or liquid biodegradable polymers have shown unique advantages as injectable matrices for sustained release of a wide range of drugs. Here we report the design, synthesis, and characterization of a new low-melt liquid copolymer based on poly(ε-caprolactone) (PCL) and establish its utility as a versatile delivery platform. **Methods**: The copolymer, mPA20, consisting of short PCL blocks connected via acid-labile acetal linkages, was synthesized using a one-pot reaction and its properties were comprehensively characterized. **Results**: mPA20 is an amorphous, injectable liquid at physiological temperature and can undergo pH-sensitive hydrolytic degradation. mPA20 bearing methacrylate end groups can be photo-crosslinked into solid matrices with tunable mechanical properties. A hydrophobic fluorophore, Nile Red (NR), was solubilized in mPA20 without any solvent. Sustained release of NR into aqueous medium was achieved using mPA20, either as an injectable liquid depot or a photo-crosslinked solid matrix. Further, mPA20 self-emulsified in water to form nanodroplets, which were subsequently photo-crosslinked into nanogels. Both the nanodroplets and nanogels mediated efficient intracellular delivery of NR with no cytotoxicity. **Conclusions**: mPA20, a new photo-crosslinkable, hydrophobic liquid copolymer with pH-sensitive degradability, is highly adaptable as either an injectable or implantable depot or nanoscale carrier for the controlled release and intracellular delivery of poorly soluble drugs.

## 1. Introduction

Hydrophobic, semi-solid or liquid polymers have gained increasing popularity in controlled drug delivery because they are easy to formulate, injectable, and compatible with a broad range of drugs from small molecules to biologics [1,2,3]. Most polymer excipients are solids and often require high temperature or potentially toxic solvents to process. In contrast, semi-solid polymers are fluidic enough to be mixed with drugs under mild conditions to create injectable dosage forms. One example is the poly(ortho esters) (POEs), first developed and reported decades ago by Heller and colleagues [4,5]. There are four generations of POEs containing cyclic structures and ortho ester linkages in the polymer backbone [6]. The most successful generation, POE IV, is synthesized from a diketene acetal and diols through a polyaddition reaction. The variety of the diol monomers allows the tuning of polymer properties including glass transition temperature and mechanical stiffness. A special oligo(glycolic acid) diol forms the so-called latent acid unit that controls the rate of polymer degradation through acid-catalyzed hydrolysis of the ortho ester linkages. Semi-solid POEs have been used in local injectable drug formulation to treat periodontal pain [7] and eye diseases [8], and incorporated in two FDA-approved drug products (Sustol^®^ and Zynrelef^®^) [9,10]. These polymers have low molecular weight and contain hydrolysable ortho ester bonds in the hydrophobic backbone. They undergo surface erosion in water and do not produce acidic byproducts, which is a significant advantage for the delivery of acid-sensitive fragile drug molecules [5]. The viscosity of these polymers can be further tuned by using plasticizers such as short-chain polyethylene glycol or biocompatible organic solvents, to allow injection using common-sized needles. However, poor storage stability, batch-to-batch variation, and difficulties with scalable manufacturing have limited the clinical translation of the POE-based drug formulations. In another example, Amsden and colleagues have developed low-molecular-weight aliphatic polycarbonates and reported their use as injectable depots for sustained release of proteins [1,11]. Recent studies show that because these polymers were viscous liquids in the physiological environment, they caused only moderate irritation of the tissue near the injection site and did not elicit serious immune response in vivo [11,12]. However, as viscous liquids, these polymers are not stiff enough to bear a load and cannot resist physical deformation, and therefore, are not appropriate for use as controlled release scaffolds in musculoskeletal tissue engineering applications.

Poly(ε-caprolactone) (PCL) is an extensively studied biodegradable polymer [13,14,15,16] and is widely used in controlled drug delivery [17] and tissue engineering [18]. PCL can be processed into many physical forms, such as micro and nanoparticles, electrospun fibers, films, meshes, porous 3D scaffolds, and can be modified through copolymerization or blending [19]. At room and physiological temperatures, PCL with an average molecular weight exceeding 2 kDa is a semi-crystalline (~50%) and mechanically stiff solid [20]. Its processing requires an organic solvent or a high temperature, both of which could damage fragile bioactive cargos. On the other hand, low-molecular-weight PCL is a viscous fluid, but it lacks mechanical robustness and degrades too quickly for most medical applications [21]. Further, the rate of hydrolytic degradation of PCL homopolymers is slow, insensitive to mild pH changes and not readily tunable [13]. Nonetheless, PCL is frequently used as a building block [22,23] to construct various block or graft copolymers that form cell-targeted nanoparticles [24,25] or in situ hydrogels for stimuli-responsive drug release [26,27] or tissue repair [28,29].

Previously we synthesized an amphiphilic liquid copolymer consisting of equal numbers of PCL and polyethylene glycol (PEG) segments that formed pH-degradable thermosensitive nanodroplets in water [30]. Here we use a similar design strategy to create a predominantly hydrophobic liquid copolymer using only PCL segments connected via acid-labile acetal linkers and explore its potential for delivering poorly water-soluble compounds. This novel copolymer also contains methacrylate end groups, allowing for its photo-crosslinking into solid matrices in situ. In this report, we describe the synthesis and comprehensive characterization of this PCL-based photo-crosslinkable liquid copolymer (code-name mPA20), aiming to showcase its versatility both as a macroscopic depot for localized drug release and as nanoscale particulates for systemic administration and intracellular delivery.

## 2. Materials and Methods

### 2.1. Materials

Poly(ε-caprolactone) (PCL) diol (average M_n_ = 530 and 2000), tri(ethylene glycol) divinyl ether (TEGDE), *para*-toluenesulfonic acid monohydrate (*p*TSA), anhydrous ethyl acetate (aEA), dimethyl sulfoxide (DMSO), tetrahydrofuran (THF), dichloromethane (DCM), basic activated alumina, sodium sulfate, Nile Red (NR), photoinitiators (PI) diphenyl(2,4,6-trimethylbenzoyl)phosphine oxide (TPO), and 2-hydroxy-2-methylpropiophenone (Irgacure 2959) were purchased from Sigma-Aldrich (St. Louis, MO, USA). 2-Hydroxyethyl methacrylate (HEMA) was purchased from Oakwood Products Inc. (Estill, SC, USA) PCL diol was dried under vacuum at room temperature for at least overnight before use. HEMA was run through a silica column to remove stabilizer before use. Other chemicals were used as received.

### 2.2. One-Pot Synthesis of mPA20

Vacuum-dried PCL diol (30 g) was dissolved in aEA, dried over sodium sulfate, and added to a reaction flask under an Argon atmosphere and stirring at 360 rpm. TEGDE at 110% molar equivalent to PCL diol was then added to the flask and stirred for at least 5 min. The catalyst, *p*TSA, at 1 wt % of TEGDE, was dissolved in 5 mL of aEA and slowly added to the solution. Polymerization continued under stirring for at least 4 h at room temperature, after which ~4 g of HEMA dissolved in 10 mL of aEA was added, and polymerization was allowed to continue for at least another 2 h. Alumina oxide was added to quench the reaction. Crude copolymer was filtered twice to remove alumina oxide, followed by rotary evaporation to remove the majority of aEA, then dialyzed against EA (membrane MW cutoff: 1000) for three days with solvent changes every 24 h. The purified product mPA20 was obtained after removing EA by rotary evaporation with further drying under vacuum continuing for at least two days at room temperature.

### 2.3. Polymer Characterization

^1^H NMR spectra were recorded on an AX-400 Bruker Avance III HD (Bruker Corp., Billerica, MA, USA) with SampleXpress and with samples solvated in DMSO-*d*_6_ with tetramethylsilane as an internal standard at 25 °C. Gel permeation chromatography (GPC) experiments were performed at room temperature with THF as solvent using a Thermo Separation Products (TSP) Spectra Systems AS1000 autosampler (Thermo Scientific, Waltham, MA, USA) equipped with three 5-mm Phenomenex Phenogel columns (Torrance, CA, USA), a Waters 515 pump (Waters Corp., Milford, MA, USA), and a Waters 2410 differential refractive index detector (Waters Corp., Milford, MA, USA). The column system was calibrated with a set of monodisperse polystyrene standards using HPLC-grade THF (Sigma-Aldrich, St. Louis, MO, USA) as a carrier solvent with a flow rate of 1 mL/min at 30 °C. Rheometry data were collected on a TA Instruments Discover Series Hybrid Rheometer 3 (Eden Prairie, MN, USA) using 8 mm parallel plates with a gap of 1 mm between plates. Temperature of the samples was maintained between 20 °C to 80 °C with a circulating water-cooling system. For Differential Scanning Calorimetry (DSC), samples were sealed in Tzero hermetic aluminum pans (TA Instruments, Eden Prairie, MN, USA) and scanned from 40 °C to −100 °C to 100 °C at a rate of 10 °C/min on a TA Q1000 calorimeter (TA Instruments, Eden Prairie, MN, USA) purged with nitrogen. Average size of polymer nanoparticles in water was determined by dynamic light scattering using a Brookhaven Instruments 90Plus Particle Size Analyzer (Nashua, NH, USA), as described previously [30].

### 2.4. Polymer Degradation Kinetics

The hydrolytic degradation profile of mPA20 was determined based on its ^1^H NMR spectra, using a method reported previously [30]. Briefly, polymer samples were dissolved in 90% DMSO-d_6_ and 10% D_2_O, with the D_2_O adjusted to pH 5.1 or 7.4, incubated at 37 °C or 60 °C, and their NMR spectra were acquired at various time points. The degree of hydrolytic degradation was calculated as a ratio of the integrals between the acid-labile acetal proton and methylene protons in the mPA backbone.

### 2.5. Photo-Crosslinking and Quantification of Gel Fraction

The PI blend consisting of 0.5% *w*/*w* TPO and 0.5% *w*/*w* Irgacure 2959 was added to mPA20 at 0.5 wt % each without solvent and mixed for 15 to 30 min in a Bransonic^®^ Ultrasonic Cleaner bath (Emerson, St. Louis, MO, USA), followed by an overnight rest at room temperature to ensure complete solubilization of the PI blend. Using a positive displacement pipette, 55 μL aliquots of mPA20/PI mixture was transferred to the space between two glass slides separated with a 1.2 mm silicon insert, followed by at least 45 s of UV irradiation using a UVGL-58 Handheld UV lamp (365 nm) (UVP, Upland, CA, USA). Round discs 8 mm in diameter and 1.2 mm in thickness were formed with an average disc weighing about 55 mg. To determine the gel fraction of crosslinked material, the discs were placed in 10 mL of 50/50 DMSO/EA and extracted under constant agitation at room temperature. After 12 and 24 h, the solvent was filtered out, and the discs were vacuum-dried at 40 °C before being weighed again. The solvent extraction continued until the dry weight of the discs ceased to change. The gel fraction was calculated as the dry weight ratio in percentage of the discs before and after solvent extraction.

### 2.6. Mechanical Compression Test

Mechanical behavior of the photo-crosslinked mPA20 discs was characterized using a TestResources 250 LB Actuator (Shakopee, MN, USA), oriented vertically with a 1 lb load cell. Negative strain was applied at a rate of 3 mm/min with a force limit of 4 N on a 5-N load cell. The point of zero strain was determined by a minimum running average of 0.05 N over the last 5 log points at a logging rate of 10/s and used as the starting position of the compression. Compressive modulus was determined with samples in triplicate after multiple cycles per sample.

### 2.7. Accelerated Polymer Erosion Test

Discs of crosslinked mPA20 were immersed in 3 mL of aqueous buffers (pH 7, 5 and 3), placed in 5 mL screw-capped glass scintillation vials and shaken at 180 rpm at 60 °C in a New Brunswick Scientific Excella E24 Incubator/Shaker (Edison, NJ, USA) for 3 days. By the end of day 3, the samples were removed and photographed to document any changes in material morphology.

### 2.8. NR Release Kinetics

Photo-crosslinked mPA20 discs or un-crosslinked liquid mPA20 of equivalent mass containing 0.1 wt % NR (~55 μg) were immersed in 3 mL of either PBS (pH 7.4) or sodium acetate buffer (pH 5.1) containing 2% *w*/*v* Tween 80 in a 5 mL screw-capped glass scintillation vial and shaken at 120 rpm at 37 °C in a New Brunswick Scientific Excella E24 Incubator/Shaker. The solubility of NR in 2% Tween 80 was determined to be greater than 10 μg/mL. We estimated that about 10% of the total NR loaded was released at each sampling time point, which would reach a concentration of ~1.7 μg/mL, much lower than the NR solubility in release medium (10 μg/mL); therefore, with the addition of Tween 80 and the volume of the buffers, the sink condition was consistently maintained. At each time point, 3 mL of the supernatant was removed from each sample, freeze-dried, and then mixed with DMSO to dissolve the released NR, which was then quantified by fluorimetry using a Biotek Cytation 3 fluorescence plate-reader (Winooski, VT, USA) at 515/585 nm for excitation/emission, in triplicate per sample. NR concentration was calculated based on a standard curve generated from serially diluted samples of known quantities of NR in DMSO containing 2% Tween 80.

### 2.9. Preparation of Nanodroplets and Subsequent Photo-Crosslinking to Produce Nanogels

Nanodroplets of un-crosslinked mPA20 were prepared by dispersing the liquid copolymer into 3 mL of DI water at 0.1, 0.5, and 1 mg/mL and sonicated using a Sonics Vibra Cell Ultrasonic Processor VCX130PB (Sonics & Materials Inc., Newtown, CT, USA) at 75% amplitude, delivering different levels of total energy ranging from 100 to 500 J by adjusting the duration of sonication. Samples were kept on ice during sonication to prevent over-heating. To prepare photo-crosslinked nanogels, the nanodroplet aqueous suspension was mixed with the PI blend and exposed to UV irradiation for at least 60 s using a UVGL-58 Handheld UV lamp (365 nm). Both the un-crosslinked and crosslinked samples were kept at 37 °C and colloidal stability was assessed by dynamic light scattering.

### 2.10. Intracellular Uptake and Cytotoxicity Assay

To assess cellular uptake, human pancreatic adenocarcinoma (AsPC-1) cells (ATCC) were exposed to NR-loaded mPA20 nanodroplets (un-crosslinked) or nanogels (photo-crosslinked) for 24 h. AsPC-1 cells were cultured in RPMI 1640 (10% Fetal Bovine Serum, 1% Penicillin/Streptomycin) (Thermo Fisher, Waltham, MA, USA) from thaw for 72 h, with a media change at 48 h. At 72 h, cells were lifted with 0.05% Trypsin-EDTA and seeded in a 12-well plate at 40,000 cells/well to result in 80% confluency after 24 h. NR (1 mg) was loaded into 100 mL of mPA20, emulsified in water at 1 mg polymer/mL, sonicated at 500 J and UV crosslinked or left un-crosslinked, and filtered through 0.45-µm Nylon filter (Thermo Fisher, Waltham, MA, USA). To each well of cells were added 20 µL of filtered crosslinked nanodroplets, un-crosslinked nanodroplets, or free NR in DMSO (without polymer) and incubated for 24 h. The cells were then washed twice with PBS, fixed with 4 *w*/*v* % paraformaldehyde (Thermo Fisher, Waltham, MA, USA), stained with Hoechst 33342 (5 μg/mL) to visualize the nucleus, and imaged by fluorescence microscopy. Intracellular uptake was quantified using ImageJ (Version 1.54k) and represented as the average fluorescence intensity (pixel area) per cell.

To assess cytotoxicity, murine fibroblasts (NIH 3T3) (ATCC) were cultured in DMEM (10% Fetal Bovine Serum, 1% Penicillin/Streptomycin) from thaw for 72 h, with a media change at 48 h. At 72 h, cells were lifted with 0.05% Trypsin-EDTA and seeded at 5000 cells/well in a 96-well plate. After 24 h, the cells were incubated with 50–1000 µg/mL of mPA20 nanodroplets for 24 h, and cell viability was quantified with an MTS assay kit (Abcam, Cambridge, UK) following instructions from the manufacturer. Untreated cells were used as a positive control (100% viability).

## 3. Results

### 3.1. Polymer Synthesis and Characterization

**Scheme 1:**
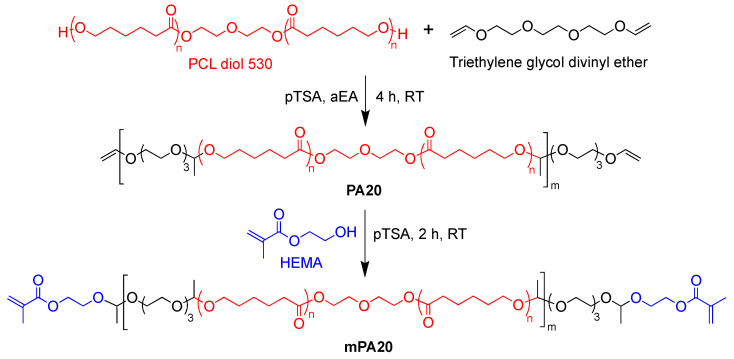
One-pot synthesis of the photo-crosslinkable liquid copolymer mPA20.

To synthesize mPA20, short chains of PCL diol (M_n_~530) were reacted with a divinyl ether (TEGDE) via acid-catalyzed polyaddition followed by end-capping with HEMA in a one-pot reaction (Scheme 1). The initial feed molar ratio between PCL and TEGDE was 1:1.1. The slight excess of divinyl ether was expected to result in a telechilic intermediate multi-block copolymer (PA20) with predominantly vinyl ether end groups. After four hours, HEMA was added to the reaction, producing the methacrylate-end-capped PA20 (mPA20) (Scheme 1). Finally, the crude product was purified by dialysis to remove any residual small molecules including unreacted TEGDE, HEMA, and the catalyst (*p*TSA).

**Figure 1:**
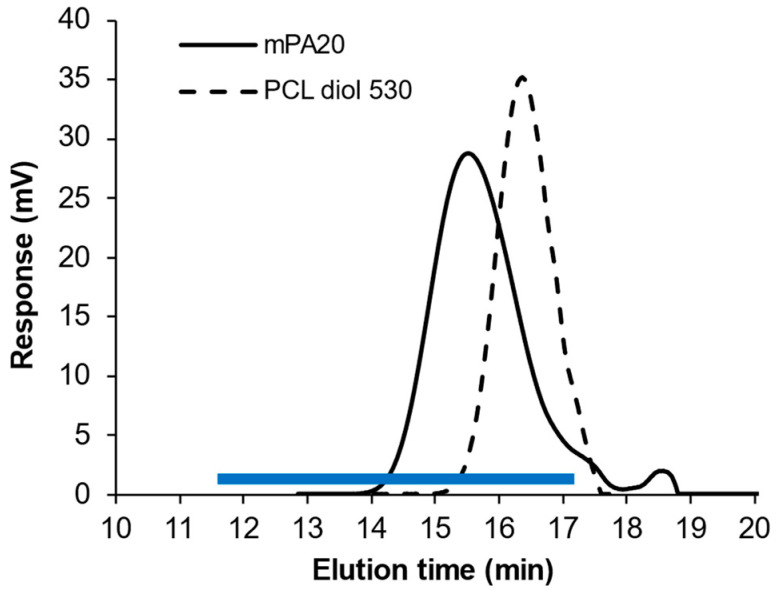
GPC profiles of the photo-crosslinkable liquid copolymer mPA20 and the starting material PCL diol 530. The blue line indicates the linear range of resolution of the size exclusion column. Mobile phase: THF, flow rate: 1 mL/min.

**Figure 2:**
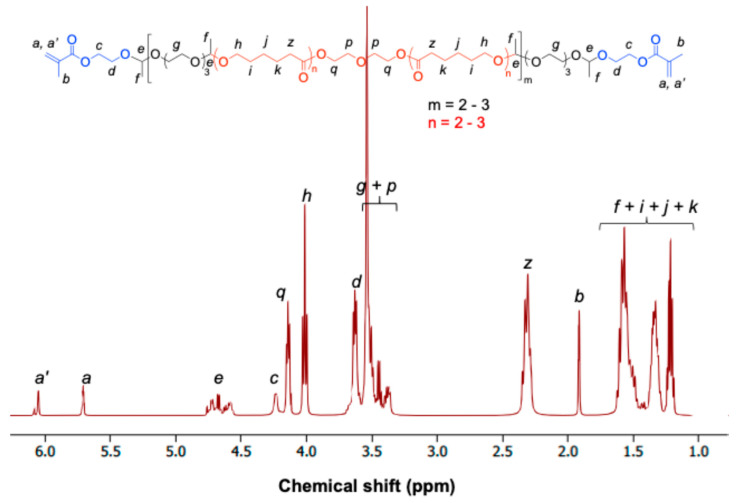
^1^H NMR (400 Hz) spectrum of mPA20 in DMSO-d_6_. The chemical structure of the di-methacrylated mPA20 is shown along with proton peak assignments. Lowercase letters refer to specific proton peaks and their corresponding protons in the chemical structure of mPA20. The degree of methacrylation was calculated to be 86%.

Successful synthesis of mPA20 was verified by GPC and ^1^H NMR. GPC analysis of the purified mPA20 showed a single peak with an elution time significantly shorter than the starting material PCL diol 530, indicating that PCL 530 was coupled by the linker to form mPA20 with a much longer chain length (Figure 1). The apparent average M_n_ of mPA20 was calculated to be approximately 2800 with a dispersity (Đ) value of 1.44. The average number of PCL blocks in mPA20 (m, Scheme 1) was calculated to be approximately 2.5. Proton NMR confirmed the presence of the acetal bond by peak e (4.65 ppm) and of the methacrylate group by peaks a and a’ (5.67 and 6.02 ppm, respectively) (Figure 2). Signals from the vinyl ether protons were not found, indicating that there was no unreacted vinyl ether group in the product. Signals from the HEMA hydroxyl proton were also not found, suggesting that excess unreacted HEMA was completely removed from the product through dialysis. In theory, methacrylation of 100% of the end groups would produce a b/z peak area ratio of 0.25. The actual b/z ratio was found to be 0.21; therefore, the degree of methacrylation was calculated to be 86%.

### 3.2. mPA20 Is an Amorphous, Low-Melt, Viscous Newtonian Liquid

**Figure 3:**
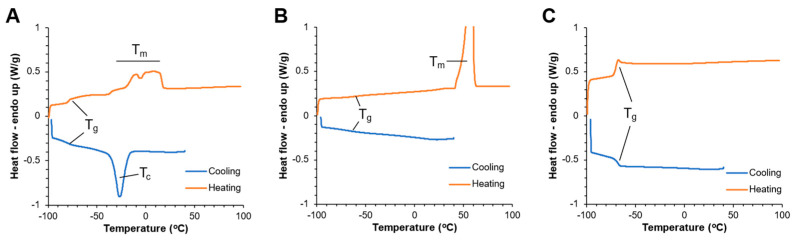
DSC analysis of (**A**) PCL 530, (**B**) PCL 2000, and (**C**) mPA20 (apparent M_n_ = 2800).

DSC analysis found that PCL 530 crystallized upon cooling followed by melting when heated to around 0 °C (Figure 3A). PCL 2000 is highly crystalline with a distinct melting event at around 50~65 °C (Figure 3B). However, mPA20 did not crystallize or melt at any temperature and remained completely amorphous (Figure 3C). The only discernable thermal event for mPA20 over the entire temperature range (−100 °C to 100 °C) was a glass transition with an apparent T_g_ of −70 °C, after which the polymer remained a viscous liquid (Figure 3C). This is attributed to the triethylene glycol acetal linkers disrupting the molecular regularity of the mPA20 backbone and limiting the mobility of the PCL blocks, thereby effectively suppressing crystallization.

**Figure 4:**
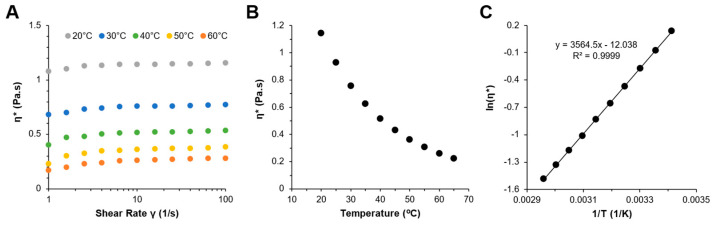
Rheological characterization of mPA20. (**A**) Complex viscosity η* as a function of shear rate γ measured at various temperatures; (**B**) Temperature dependence of η* measured at 10/s shear rate; (**C**) Linear correlation between ln(η*) and 1/T in K.

The solvent-free mPA20 at room temperature was a viscous fluid with a complex viscosity (η*) of about 1 Pa.s, making it easy to inject through regular gauge needles. The η* of mPA20 was not dependent on the shear rate (γ) at any of the temperatures tested (Figure 4A), but it decreased as the temperature increased (Figure 4B), a typical behavior of a Newtonian fluid [11]. The temperature dependence of η* followed the Arrhenius relationship as shown by a perfect linear fit between ln(η*) and the inverse of temperature (T) in Kelvin (Figure 4C).

### 3.3. Hydrolytic Degradation of mPA20

**Scheme 2:**
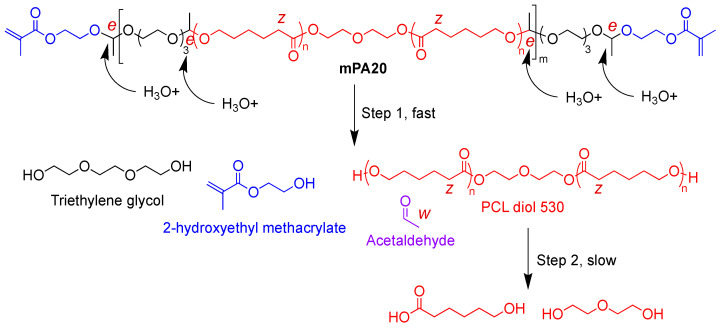
The postulated two-step pathway of hydrolytic degradation of mPA20. Signals of the characteristic protons used in the NMR analysis to monitor the progression of hydrolysis are indicated by small letters in italic (*e*, *z*, and *w*, see Figure 5 for reference).

In theory, the acetal linkage in the backbone of mPA20 is expected to hydrolyze in aqueous media. The rate of hydrolysis should be faster at mildly acidic pH than neutral or alkaline pH. Higher temperature will accelerate the hydrolysis as well. Therefore, we postulate that the hydrolysis of mPA20 may follow a two-step process (Scheme 2). Step 1 is a fast step, through which the acetal bond is cleaved by water, producing the water-soluble triethylene glycol, 2-hydroxyethyl methacrylate (from the polymer end groups), acetaldehyde, and the water-insoluble PCL diol 530. Step 2 involves the much slower hydrolysis of the PCL diol 530 to its water-soluble small-molecule building blocks, mainly 6-hydroxylcaproic acid (Scheme 2).

**Figure 5:**
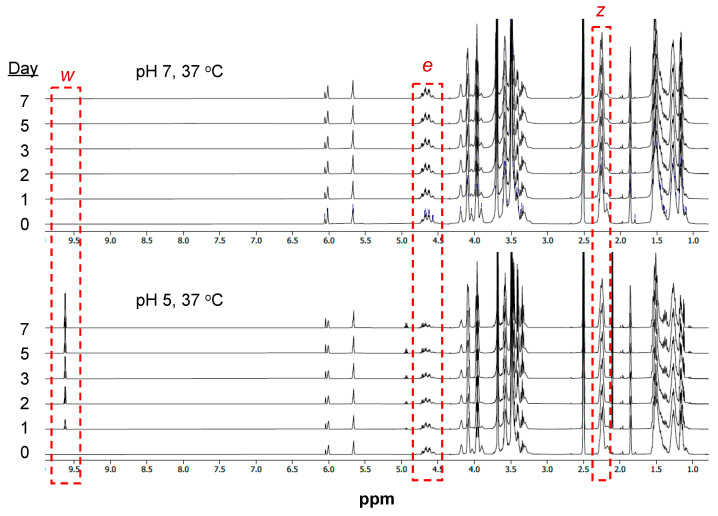
Acid-labile hydrolysis of mPA20 in 90% DMSO-d_6_/D_2_O at 37 °C analyzed using ^1^H NMR at 400 Hz. Characteristic proton signals are highlighted in red.

**Figure 6:**
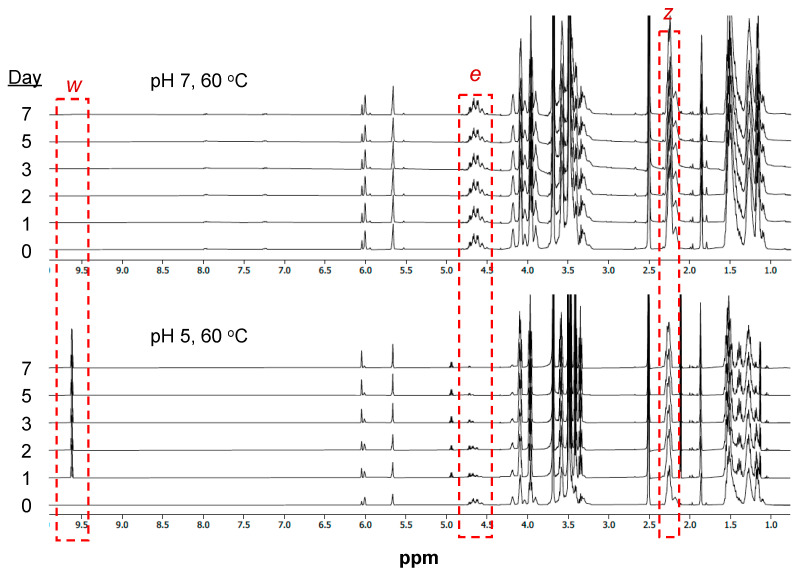
Accelerated acid-labile hydrolysis of mPA20 in 90% DMSO-d_6_/D_2_O at 60 °C analyzed using ^1^H NMR at 400 Hz. Characteristic proton signals are highlighted in red.

**Figure 7:**
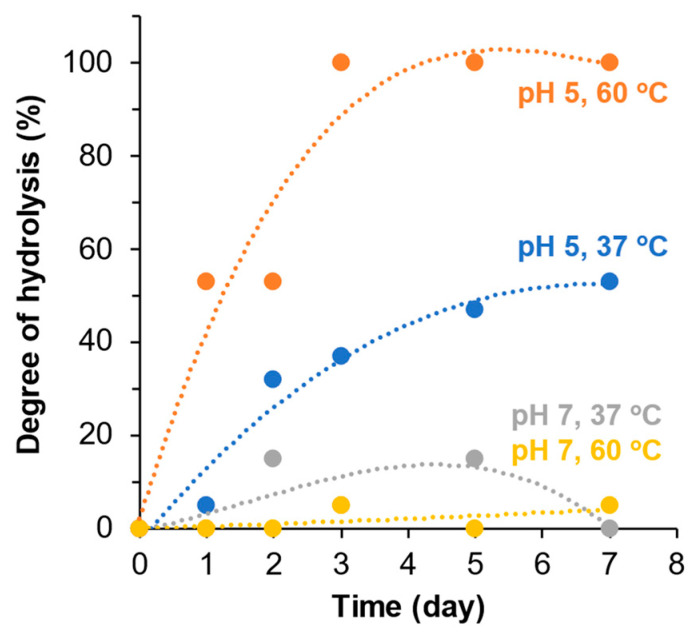
Kinetics of hydrolytic degradation of mPA20 via the cleavage of acetal linkages in the polymer backbone. The degree of hydrolysis is determined by ^1^H NMR at different pHs (7 and 5) and temperatures (37 and 60 °C).

To elucidate the hydrolytic mechanism of mPA20, we recorded ^1^H NMR spectra of mPA20 dissolved in a DMSO/water mixed solvent and exposed to different pHs and temperatures over time. This method has been used by us previously to study the hydrolysis of an amphiphilic liquid copolymer of PCL and PEG [30]. The peak area of the characteristic protons of the acetal linkage (O-C*H*(CH_3_)-O, *e*), the PCL block (O=C-C*H*_2_-, *z*), and a small-molecule hydrolysis product (acetaldehyde, *w*) were calculated. At neutral pH 7 and physiological temperature (37 °C), changes to the *e* and *z* peaks were negligible, and the *w* signal was not detected, indicating that mPA20 remained stable over 7 days (Figure 5, top panel). However, at a mildly acidic pH 5 and 37 °C, there was a marked decrease in the acetal proton signal *e* and a growing intensity of peak *w* over time, while the PCL proton z remained unchanged, suggesting that accelerated hydrolysis of the acetal linkage in the polymer backbone occurred (Figure 5, bottom panel). Raising the temperature from 37 °C to 60 °C did not change the NMR spectra or any characteristic peaks of mPA20 at pH 7 over 7 days (Figure 6, top panel), but at pH 5, it significantly accelerated the cleavage of the acetal bond, manifested by decreasing signal *e* and increasing signal *z* (Figure 6, bottom panel). The pH and temperature dependence of the kinetic profiles of mPA20 hydrolysis as determined by NMR is quantified and summarized in Figure 7, showing clearly that mildly acidic pH and elevated temperature accelerate the hydrolysis. It is important to note that here, the “degree of hydrolysis” is computed based on the decrease in the acetal proton signal *e* in relation to the PCL proton signal *z*, thus reflecting only the Step 1 hydrolysis of mPA20 through the cleavage of the backbone acetal bond. The Step 2 hydrolysis of the residual PCL 530 is too slow to be captured by our study over the 7-day period.

### 3.4. Photo-Crosslinking of mPA20

**Figure 8:**
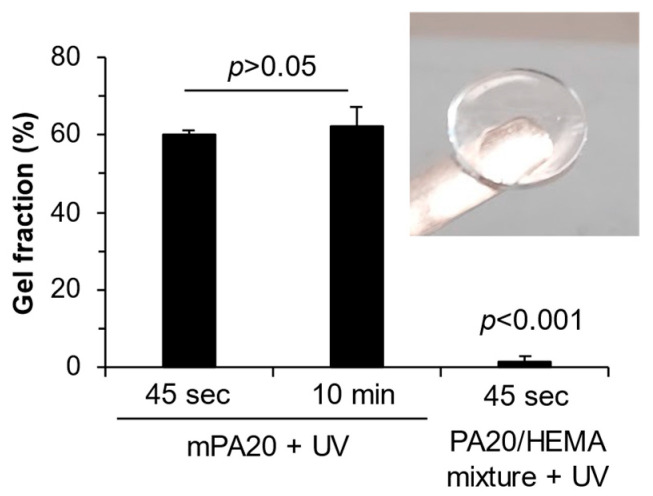
Gel fraction of the UV-crosslinked mPA20 discs. The duration of irradiation of 45 s or 10 min did not change the gel fraction significantly. A simple mixture of PA20 and HEMA did not crosslink under the same UV irradiation. Mean +/− SD, *t*-test (unpaired), *p* > 0.05: not significant. Inset photo shows a crosslinked mPA20 disc.

Using a common photoinitiator blend [31], the liquid copolymer mPA20 was crosslinked without solvent by irradiating with UV at 365 nm. Forty-five seconds of irradiation at room temperature was sufficient to transform mPA20 from a viscous liquid to a solid resin. Discs of crosslinked mPA20 (Figure 8, inset photo) appeared optically clear and macroscopically homogeneous. We also synthesized PA20, an intermediate of mPA20, without the methacrylate end groups, blended it with HEMA without solvent at the same feed ratio as was used in the synthesis of mPA20, and exposed the mixture to UV in the presence of photoinitiators. We found that this simple mixture of PA20 and HEMA was unable to solidify even after multiple and prolonged irradiation (Figure 8), confirming that the crosslinked product is indeed the result of covalently incorporated methacrylate end groups of mPA20 (Scheme 1).

The gel fraction of the photo-crosslinked discs was measured to be around 60% by weight. A much longer duration of UV irradiation did not increase the gel point significantly beyond 60% (Figure 8), due to the limited kinetic chain mobility of the bulk mPA20 liquid copolymer in the absence of solvent. As expected, a simple PA20/HEMA mixture did not crosslink and had a negligible gel fraction (Figure 8). Taken together, we conclude that the molecular structure of the photo-crosslinked mPA20 can be best described as a semi-interpenetrating network (semi-IPN) with maximal 60% crosslinked polymer chains interwoven with 40% presumably linear chains.

### 3.5. Mechanical Properties of the Photo-Crosslinked mPA20

**Figure 9:**
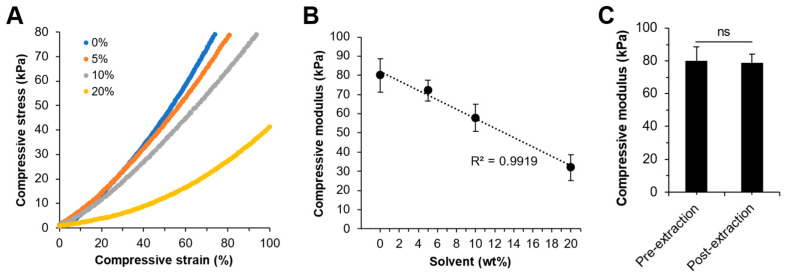
Mechanical properties of the photo-crosslinked mPA20 discs. (**A**) Compressive stress–strain profiles and (**B**) compressive moduli of samples photo-crosslinked in the presence of various amount of solvent (ethyl acetate). (**C**) Compressive moduli of crosslinked samples before and after solvent extraction. Data represented by mean +/− SD. ns: not significant (*p* > 0.05, paired *t*-test).

Photo-crosslinking the mPA20 liquid copolymer under solvent-free conditions produced a solid material that was highly elastic and mechanically robust. A compression test shows that the solvent-free crosslinked product underwent a large elastic deformation, achieving a compressive stress of at least 80 kPa and a compressive strain of at least 70% (Figure 9A). Even higher stress and strain was attainable but not recorded due to the limited capacity of the load cell. The compression test was repeated for several cycles and identical stress–strain curves were obtained. There was no hysteresis nor any sign of irreversible plastic deformation, showcasing the robustness of the material and its ability to undergo large elastic deformation. While being a solvent-free system is a key feature of the mPA20 liquid copolymer, we further found that by including a solvent (such as ethyl acetate) during the photo-crosslinking process, the mechanical behavior of the crosslinked products can be tuned. Increasing the amount of solvent from 5 to 10% improved the deformability of the crosslinked product significantly, resulting in maximal compressive strains of 80% and 90%, respectively, while maintaining the same compressive stress of 80 kPa (Figure 9A). There is a negative linear correlation between the compressive modulus of the crosslinked product and the amount of solvent from 80 ± 9 kPa solvent-free to 72 ± 6 kPa, 58 ± 7 kPa, and 32 ± 7 kPa at 5, 10, and 20% *w*/*w* solvent, respectively (Figure 9B). Finally, there was no statistical difference between the compressive moduli of the samples before and after solvent extraction, which removed any un-crosslinked linear chains (i.e., the ‘sol’ fraction), suggesting that the crosslinked network (i.e., the ‘gel’ fraction) was solely responsible for the mechanical behavior of the semi-IPN (Figure 9C).

### 3.6. Accelerated Erosion of Photo-Crosslinked mPA20

**Figure 10:**
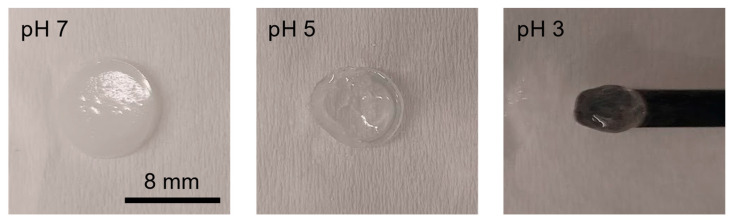
Accelerated erosion test of the photo-crosslinked mPA20 discs in aqueous buffers of different pHs. Discs in triplicate were immersed in pH 7, 5, and 3 phosphate buffers and incubated at 60 °C, 180 rpm for 3 days. A spatula was used for convenient handling of the sample at pH 3 due to extensive disintegration of the material.

Discs of crosslinked mPA20 were hydrolyzed at elevated temperature (60 °C) and various pHs from neutral to acidic. By the end of day 3, the discs were very stable under physiologic pH without any visible signs of erosion (Figure 10). However, discs at pH 5 became smaller and were visibly eroded. The erosion of the discs was most prominent at pH 3. This pH-dependent erosion behavior is consistent with the pH-dependent hydrolytic degradation of mPA20 shown in Figure 7 caused by cleaving the backbone acetal bonds (Scheme 2, Step 1), whereas the erosion at extreme acidic pH is likely accompanied by the hydrolysis of both the backbone acetal and the PCL ester bonds (Scheme 2, Steps 1 and 2).

### 3.7. Loading and Release Kinetics of a Poorly Water-Soluble Compound

**Figure 11:**
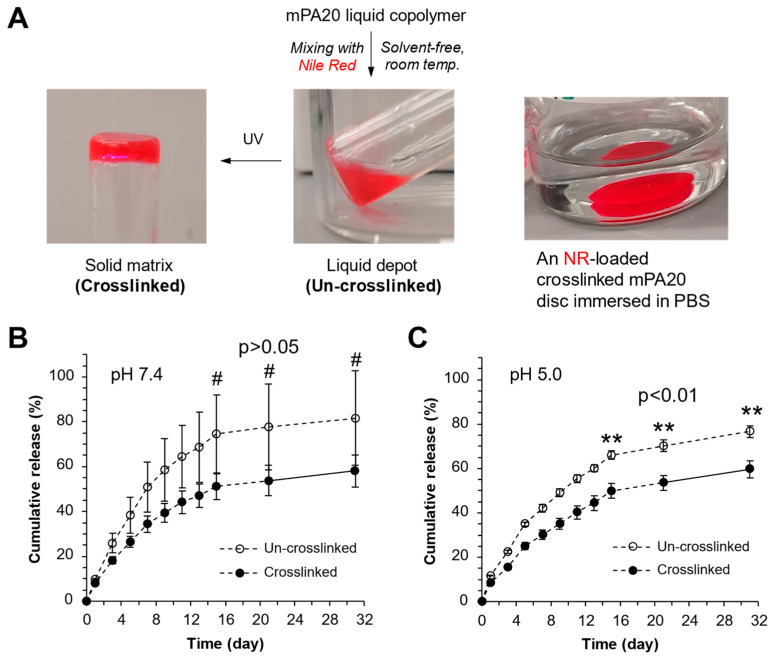
Loading and release of NR from un-crosslinked mPA20 viscous liquid and crosslinked mPA20 solid matrix. (**A**) The process of NR loading (0.1 wt %) and sample preparation; an NR-loaded mPA20 disc immersed in PBS for release studies. (**B**,**C**) Cumulative release of NR over time at pH 7.4 or 5.0, 37 °C. Data points are mean +/− SD, n = 3. Unpaired *t*-test, #: *p* > 0.05, **: *p* < 0.01.

NR, a hydrophobic, water-insoluble fluorophore, was used as a model compound. First, NR was loaded into the un-crosslinked liquid mPA20 by simple admixing without needing any solvent, because mPA20 is a low-melt liquid at room temperature and it alone acts as an effective solvent. Next, the NR-loaded mPA20 was added to PBS to quantify NR release over time. The viscous, hydrophobic mPA20 was not miscible with the aqueous buffer and remained phase separated. In parallel the NR-loaded mPA20 was photo-crosslinked into a solid matrix and then immersed in PBS to quantify the release of NR (Figure 11A). The sink condition was maintained during the entire period of the experiments. Over the course of thirty days, the NR release kinetics for both un-crosslinked and crosslinked mPA20 followed a similar two-phase profile: an initial phase of fast release lasting approximately 15 days followed by a second phase of much slower release (Figure 11B,C). After day 15, more NR was released from un-crosslinked mPA20 than crosslinked mPA20 regardless of the pH of the media. Such a difference was not statistically significant for pH 7 (Figure 11B) due to large variations within the “un-crosslinked” data set, but was statistically significant for pH 5 (*p* < 0.01, unpaired *t*-test) (Figure 11C). It appears that pH alone did not affect the release kinetics.

### 3.8. mPA20 Self-Emulsified in Water to Form Nanodroplets That Were Subsequently Photo-Crosslinked into Nanogels

**Figure 12:**
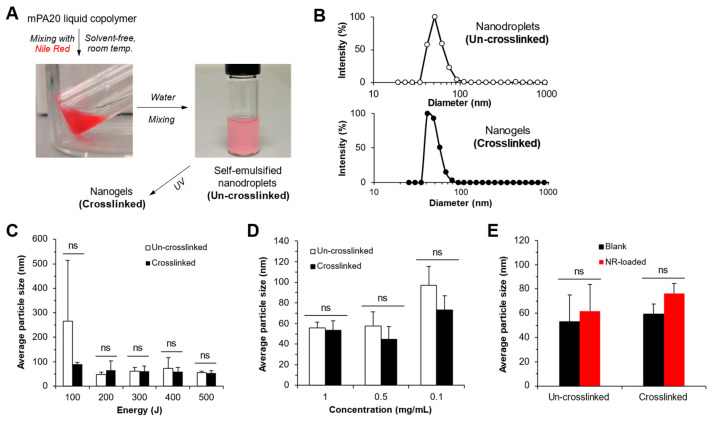
Self-emulsification of mPA20 to form nanodroplets in water and the subsequent photo-crosslinking to form nanogels. (**A**) Preparation of the un-crosslinked nanodroplets and crosslinked nanogels. (**B**) Particle size distribution by dynamic light scattering. Sonication energy output: 500 J. mPA20 concentration: 1 mg/mL. (**C**) The effect of sonication energy output level on particle size. (**D**) The effect of mPA20 concentration on particle size. (**E**) The effect of NR loading on particle size. (**C**–**E**) mean +/− SD, Unpaired *t*-test, ns: not significant (*p* > 0.05).

When the mPA20 liquid copolymer (with or without NR loading) was added to water at 1 mg/mL and mixed using a sonication probe at 500 J energy output for around 30 s, a homogenous emulsion was formed (Figure 12A). The emulsion appeared to be stable for several days without visible phase separation or precipitation. DLS revealed that the emulsion consisted of a single population of nanodroplets with an average diameter of 55.7 ± 3.2 nm (Figure 12B). We then subjected the emulsion to the same UV treatment that crosslinked the bulk mPA20, resulting in the crosslinking of the nanodroplets into nanogels (Figure 12A), which had a single population of 48.8 ± 2.4 nm in average diameter (Figure 12B). Reducing the energy output level of sonication from 500 to 200 J did not change significantly the size of the nanodroplets and nanogels, which were all smaller than 100 nm (Figure 12C). However, sonication at 100 J produced heterogenous nanodroplets with large size variation, suggesting that the energy level was not sufficiently high to disperse the liquid copolymer to form stable droplets. Interestingly, photo-crosslinking the nanodroplets after 100 J sonication resulted in small <100 nm and more homogenous nanogels (Figure 12C). Reducing the concentration of mPA20 during sonication from 1 to 0.5 to 0.1 mg/mL led to a slight increase in particle size, but there was no significant size difference between the un-crosslinked nanodroplets and crosslinked nanogels (Figure 12D). Finally, loading the NR at 0.1 wt % had no impact on the size of un-crosslinked or crosslinked particles (Figure 12E).

**Figure 13:**
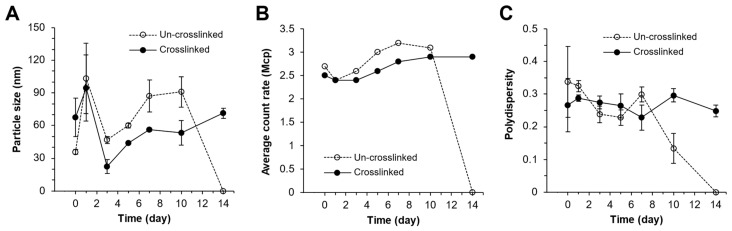
Stability of aqueous solutions of un-crosslinked nanodroplets and crosslinked nanogels assessed by dynamic light scattering in terms of time-dependent changes of particle size (**A**), average count rate (**B**) and polydispersity (**C**). Samples contained mPA20 at 1 mg/mL and were kept at 37 °C without agitation.

The stability of the mPA20 nanodroplets with or without crosslinking was further monitored for up to two weeks by DLS. The average size of both the un-crosslinked and crosslinked particles fluctuated considerably, especially during the initial 3 days, with an increase by day 2, a decrease by day 3, then a gradual increase till day 10 (Figure 13A). During this time, the average count rate (a measure of both particle size and number) and polydispersity of the particles remained steady (Figure 13B,C). Notably, the recorded values of particle size, average count rate, and polydispersity of the un-crosslinked nanodroplets dropped to zero by day 14, indicative of particle disintegration and/or aggregation (Figure 13). This observation demonstrates that photo-crosslinking greatly stabilized the mPA20 nanodroplets by converting them to nanogels.

### 3.9. Intracellular Uptake and Cytotoxicity

**Figure 14:**
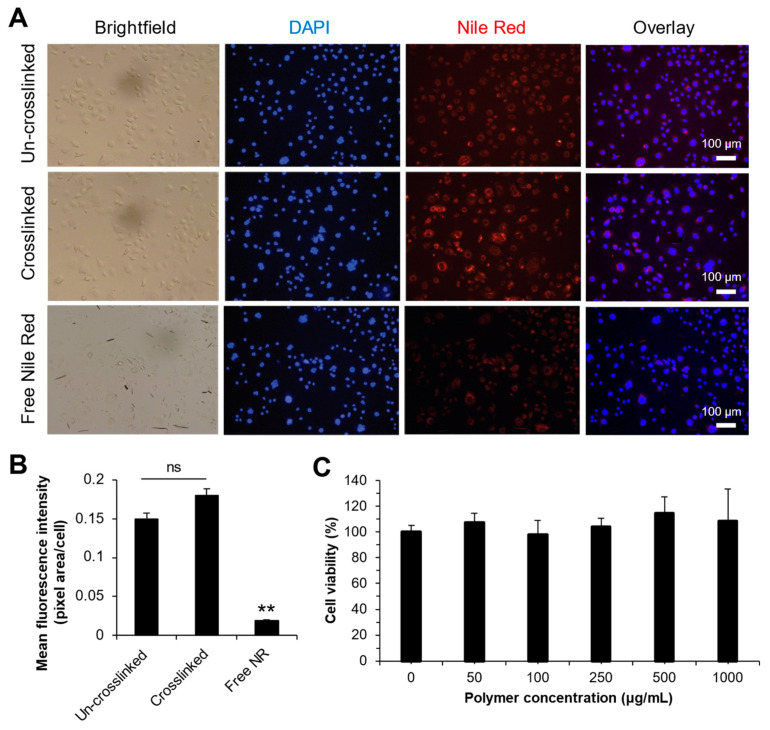
Intracellular uptake and cytotoxicity of un-crosslinked nanodroplets and crosslinked nanogels. (**A**) Representative fluorescence microscopy images of AsPC-1 cells treated for 24 h. (**B**) Quantification of the cellular uptake. (**C**) Cytotoxicity of nanodroplets to NIH 3T3 cells assessed using the MTS assay. Data points are mean +/−SD, ANOVA, ** *p* < 0.01, ns: not significant.

Fluorescence microscopy shows that NR-loaded un-crosslinked mPA20 nanodroplets and crosslinked nanogels were readily internalized by AsPC-1 human pancreatic carcinoma cells within 24 h (Figure 14A). The intracellular NR was seen primarily in the cytoplasm and not in the nucleus. The cytoplasmic NR signal appeared diffusive, rather than punctate, suggesting that the NR was not confined inside the endolysosomes. The level of intracellular uptake was similar between the two types of nanoparticles but much greater than free NR (Figure 14B). An MTS assay shows that 100% of the NIH3T3 murine fibroblasts were viable after 24 h exposure to as much as 1 mg/mL of the mPA20 nanodroplets, demonstrating that the mPA20 is nontoxic and highly compatible with cells (Figure 14C).

## 4. Discussion

### 4.1. Polymer Design, One-Pot Synthesis, and Injectability

The photo-crosslinkable liquid copolymer mPA20 is composed of short blocks of PCL connected via acid-labile acetal linkages and methacrylate end groups (Scheme 1). PCL is chosen as the main building block of the new liquid copolymer for its low glass transition and melting temperatures as well as its well-established biocompatibility [13,14,15]. The synthesis of mPA20 is accomplished through a one-pot process, starting with the *p*-TSA-catalyzed polyaddition reaction between a diol and divinyl ether. This reaction has been used in the past to synthesize water-soluble polyacetals [32,33] and PEG-polyacetal graft copolymers [34] for drug delivery. The advantages of this reaction are its simplicity, wide selection of monomers, and high efficiency under mild conditions (room temperature). As the reaction was proceeding toward completion, an excess amount of HEMA was added to cap the ends of the growing polymer chain and introduce the polymerizable methacrylate groups. We have achieved a high degree of methacrylation of 86%. Further improvement of methacrylation may be possible by optimizing the feed ratio of the initial diol and divinyl ether monomers. The acetal linker serves two purposes: (1) to disrupt the propensity of the PCL to crystallize, so that the multi-block copolymer will remain amorphous and low-melting, and (2) to enable acid-labile hydrolytic degradation of the polymer backbone. Our experimental results show that both design objectives have been achieved. DSC (Figure 3) confirms that mPA20 is low-melting and amorphous over a wide temperature range. Rheometry (Figure 4) shows that mPA20 is a Newtonian fluid, meaning that despite an M_n_ of 2500, there is little chain entanglement or chain–chain interaction. The low viscosity of mPA20 (0.5 Pa.s at 37 °C) allows for easy, solvent-free loading of drugs by gentle mixing under mild temperatures. This viscosity is also low enough for the polymer to be injected through syringe needles with ease into subcutaneous or intramuscular tissue [1]. The injectability of mPA20 compares favorably with the semisolid POE formulations of the two FDA-approved products (Sustol^®^ and Zynrelef^®^) [9,10]. No plasticizers such as PEG or potentially toxic organic solvents are necessary for mPA20. Such qualities make mPA20 an attractive material as injectable depots for the controlled release of a wide range of small molecules and biologics.

### 4.2. Acid-Labile Hydrolytic Degradation

The NMR-based hydrolysis study confirms that mPA20 does follow a two-step process as depicted in Scheme 2. A previously reported amphiphilic liquid copolymer, PA11, which is composed of a 1:1 mix of PCL and PEG blocks and acetal linkages, fully degraded by day 3 under the same conditions [30]. In comparison, mPA20 is much more hydrophobic, which explains its slower degradation rate than PA11 (Figure 7). It is well established that the hydrolytic degradation of PCL is slower than other biodegradable polyesters such as polylactide, polyglycolide, and poly(lactide-*co*-glycolide)s (PLGA) of similar molecular weight [15]. This is largely due to the greater hydrophobicity and high crystallinity of PCL. PCL hydrolysis is also insensitive to modest pH changes. By inserting acid-labile acetal linkages in between short PCL segments, we have created mPA20 as a variant of PCL capable of rapid pH-responsive hydrolysis, producing short PCL chains during degradation. After this initial fast step of hydrolysis, the short PCL chains either continue to hydrolyze to water-soluble small molecules or are phagocytosed and metabolized directly in the in vivo environment [13]. This feature allows mPA20 to be useful as an intelligent drug carrier that responds to mildly acidic pH environments typically found in highly inflamed tissues, such as a solid tumor, as well as intracellular endolysosomal vesicles. It is noted that a similar strategy was implemented recently to design polyester (including PCL) copolymers containing ortho ester linkages, which also underwent pH-sensitive degradation as a viscous liquid [35]. In comparison, mPA20 offers an important advantage of photo-crosslinking that makes it possible to transition from the liquid state to solid state through external control.

### 4.3. Photo-Crosslinking, Tunable Mechanics, and Material Erosion

We have demonstrated that the low-melt, liquid copolymer mPA20 can be transformed in situ into an elastic solid matrix by photo-crosslinking the methacrylate end groups (Figure 8). A gel fraction of 60% was observed and a higher gel fraction could not be achieved by prolonged photo-crosslinking, suggesting that the top limit of gelation was reached. The efficiency of photo-crosslinking may be improved further by obtaining macromers with a higher degree of methacrylation, but the considerable chain length of mPA20 makes photo-crosslinking the end groups intrinsically difficult. The high elasticity of the crosslinked mPA20 network is a significant improvement over other PCL-based crosslinked materials that tend to be very stiff with moduli in the GPa and MPa range [31]. Crosslinked mPA20 discs show stiffness in the tens of kPa range and can be tuned easily by varying the amount of solvent in the mPA20 liquid copolymer. The crosslinked matrices are also remarkably durable enough to withstand multiple cycles of strain greater than 65% (Figure 9). For comparison, the stiffness of adult cardiac tissue is 10–20 kPa [36] and collagenous bone tissue has a stiffness as low as 100 kPa [37]. Therefore, the crosslinked mPA20 may be an ideal material for in situ-forming drug depots and mechanically compatible cardiac or orthopedic implants, able to bear loads and deform elastically as the typical musculoskeletal tissues. Such mechanical compatibility between the mPA20 implant and surrounding tissue is expected to result in less irritation, less immune response and inflammation, and overall better biocompatibility. The crosslinked mPA20 network was quite stable at pH 7 in water. Appreciative erosion by hydrolysis was only observed under the accelerated condition of acidic pH (5 and 3) at elevated temperature (60 °C). The impact of erosion by hydrolysis on the mechanical properties of the crosslinked material warrants further study.

### 4.4. Solvent-Free Drug Loading and Mechanism of Drug Release

A key objective of our study is to demonstrate that both the un-crosslinked liquid mPA20 and the crosslinked solid mPA20 can serve as in situ depots for the controlled release of poorly soluble drugs. Over 90% of all drug candidates in the pipeline have low solubility in water [38], which seriously limits the bioavailability of these compounds. Polymer-based excipients are needed to enhance the solubility and stability and provide controlled release of poorly soluble drugs. Here we used NR, a hydrophobic insoluble fluorophore, as a model drug. Because mPA20 is a low-melt liquid, NR can be loaded in the polymer by simple admixing without requiring any solvent or heating. This is expected to reduce the overall cost of drug formulation, avoid heat-induced drug degradation, and lessen the negative impact of toxic organic solvents on the environment and on human health [39].

A couple of technical details in the release study are worth noting. To ensure a sink condition in the release medium, 2% *w*/*v* Tween 80 was included to solubilize the hydrophobic NR. The solubility of NR in the release medium supplemented with the surfactant was found to be greater than 10 μg/mL, which would allow for any burst release to be detected with 3 mL of release medium and NR loading at 0.1% *w*/*w*. Another challenge with using NR as a model drug is that its fluorescence may be subject to self-quenching when its concentration is too high or if it is poorly soluble [40]. As a workaround, we extracted and lyophilized the release medium and then resuspended the dried residue in DMSO. Because DMSO can fully solubilize NR at high concentrations, we are able to circumvent NR’s self-quenching behavior and accurately quantify the amount in the release medium.

Sustained release of NR from both un-crosslinked and crosslinked mPA20 was observed for up to a month with around 80% cumulative release (Figure 11). The remaining 20% is likely retained in the progressively more hydrophobic depot due to the increasing production of the PCL diol as a major degradation product of mPA20. Complete release of NR is expected to occur over a much longer period as the short-chain PCL degrades completely (i.e., the slower, Step 2 degradation, Scheme 2). There was no burst release with less than 6% of the payload released by day 1. Low or no burst release by mPA20 is an advantage over PLGA-type materials, because it avoids potential toxicity of drugs with a narrow therapeutic window [41].

As the release of Nile Red from the crosslinked polymers was not affected by pH, we predict that the primary mechanism of release for this system is through diffusion of the hydrophobic cargo into the surrounding environment. The initial rapid phase of NR release may also have been spurred by the infiltration of water to hydrate the hydrophilic segments of the polymer (i.e., the triethylene glycol units in the backbone). As the fast Step 1 hydrolysis took place (Scheme 2), short-chain PCL was produced and the other small-molecule products escaped and dissolved in the buffer. As a result, the drug depot might become more hydrophobic and NR release may slowdown, which is what we observed. It was anticipated that the liquid polymer depots would yield a greater release rate of NR than the crosslinked polymer, due to its lack of organized structure in the bulk and the fluidity to shed small droplets upon contact with water, as mPA20 already exhibits some degree of self-emulsifying behavior. These nanodroplets would be further stabilized by the surfactant Tween 80 in the release medium and potentially be extracted during medium exchange, thereby being counted towards the release profile. Of course, great care was taken to avoid removing any polymer vesicles large enough for the eye to see. The fluidic nature of the un-crosslinked mPA20 may allow for more deformation than the crosslinked mPA20, potentially enlarging the surface area of the polymer depot and resulting in more NR release, which is what we observed (Figure 11B,C).

### 4.5. Dynamics of the mPA20 Nanodroplets and Nanogels in Aqueous Solution

Self-emulsification of mPA20 in water did not happen spontaneously as the previously reported amphiphilic PA11 [30] and required sonication. Although the chemical structure of mPA20 is amphipathic, it is more hydrophobic (e.g., the PCL segments) than hydrophilic (e.g., triethylene glycol segments). Nonetheless, once the polymer was forced to disperse in water through sonification, the surface of the nanodroplets was sufficiently hydrophilic to stabilize against aggregation (Figure 12A). Once the energy output of the sonication reached a certain level (200 J or higher), the nanodroplet size no longer changed (Figure 12C). The polymer concentration and NR loading did not change the average particle size of the nanodroplets either (Figure 12D,E). Un-crosslinked nanodroplets and crosslinked nanogels of mPA20 exhibited interesting dynamic behavior in water. First, there was considerable fluctuation in the average particle size of both types of nanoparticles during the initial period after sonication (Figure 13A). The exact reason for this phenomenon is not clear and requires further investigation. We speculate that this might be related to the dynamic infiltration of water into the nanoparticles, causing them to swell first and then shrink as the water causes the hydrophobic PCL segments to collapse. When the hydration of nanoparticles progresses toward equilibrium, the polymer chains relax gradually, and the particle size increases again until reaching equilibrium by day 7. Second, at equilibrium (day 7 through 10), the average size of the nanogels was smaller than nanodroplets due to more restricted swelling imposed by the covalent crosslinks. Photo-crosslinking also reduces the mobility of the polymer chains so that the nanogel particles cannot fuse as easily as the highly fluidic nanodroplets. This explains the greater colloidal stability of the nanogels in water being maintained over 2 weeks, whereas the nanodroplets precipitated out by day 14.

### 4.6. Intracellular Uptake of Liquid and Solid Nanoparticles

Cellular uptake of nanoparticles is affected by many factors, including particle size, shape, charge, surface chemistry, and deformability [42]. Numerous recent studies have concluded that mechanically stiffer nanoparticles are internalized more readily by cells, in particular cancer cells, than softer nanoparticles [43]. However, cellular uptake of “very soft”, liquid nanoparticles (i.e., nanodroplets) has not been investigated in detail. The softest nanoparticles studied so far are still solids with Young’s modulus in the range of hundreds of Pa [42]. Our mPA20 liquid copolymer forms liquid nanodroplets in water and can be crosslinked into solid nanogels. These nanodroplets and nanogels are almost identical physically and chemically except for their liquid/solid state. To our surprise, we found that the uptake of NR-loaded nanodroplets and nanogels by a human cancer cell line over 24 h was not significantly different and that both the liquid and solid particles greatly enhanced the internalization of NR by these cells compared to free NR (Figure 14A,B). This is unexpected, because the prevailing paradigm would suggest that very soft, liquid nanoparticles would not be able to engage with the cellular internalization machinery nearly as effectively as stiffer solid particles. Clearly, our observation is preliminary and further investigation is necessary. Nonetheless, our study shows that the mPA20 liquid copolymer and its derivatives may be a very useful material platform to help shed light on how cells interact with liquid nanodroplets.

### 4.7. Implications for Drug Delivery

The most significant feature of mPA20 liquid copolymer is its versatility as a drug carrier of various physical forms that can be adapted for different drug delivery and controlled release applications. These physical forms are as follows: (1) solvent-free injectable liquid depot, (2) in situ curable and 3D printable depot, (3) surfactant-free nanodroplets, and (4) crosslinked nanogels. The chemical structure of mPA20 mainly consists of PCL segments, which bring considerable hydrophobicity to the construct and can solubilize hydrophobic compounds such as NR. The fluidity of the un-crosslinked mPA20 may allow it to fill body cavities of irregular shape after being injected locally into the subcutaneous or muscular tissue, thereby forming a more intimate material/tissue interface. Local injection of the liquid mPA20 may be followed by photo-crosslinking to solidify the polymer in situ. Alternatively, the liquid mPA20 may be used as an ink and 3D printed to form mechanically tunable solid constructs in vitro and then implanted in vivo. Drug-loaded mPA20 can be emulsified in water into <100 nm nanodroplets and administered systemically via intravenous injection. The stability and deformability of the nanodroplets can be further tuned by photo-crosslinking into nanogels, and both the nanodroplets and nanogels are excellent intracellular delivery vehicles for poorly soluble compounds, as demonstrated in our study.

## 5. Conclusions

In this work, we synthesized a novel PCL-based photo-crosslinkable liquid copolymer, mPA20, using a simple one-pot process. As a low-melt liquid, mPA20 can be loaded with poorly soluble compounds by simple mixing, injected as a depot, and its payload can be released over a prolonged period. Photo-crosslinking converts the liquid mPA20 to solid matrices with tunable mechanical properties. In addition, mPA20 can be emulsified in water to form nanodroplets, which can then be crosslinked into nanogels for efficient intracellular drug delivery. mPA20 is composed of biocompatible building blocks and undergoes pH-responsive hydrolytic degradation. Both mPA20 and its degradation products are not toxic to cells. Excellent biocompatibility, scalable synthesis, and versatility as an injectable, implantable drug depot as well as nanocarriers make mPA20 an attractive material platform for a broad range of drug delivery applications.

## Data Availability

The original contributions presented in the study are included in the article, further inquiries can be directed to the corresponding author.
